# Initial Effects of Differently Treated Biogas Residues from Municipal and Industrial Wastes on Spring Barley Yield Formation

**DOI:** 10.1371/journal.pone.0154232

**Published:** 2016-04-26

**Authors:** Nadia Prays, Martin Kaupenjohann

**Affiliations:** 1 Department of Bioenergy, Helmholtz Centre for Environmental Research–UFZ, Halle, Germany; 2 Department of Soil Sciences, Technische Universitat Berlin, Berlin, Germany; Chengdu Institute of Biology, CHINA

## Abstract

Soil application of biogas residues (BGRs) is important for closing nutrient cycles. This study examined the efficiency and impact on yields and yield formation of solid-liquid separated residues from biodegradable municipal and industrial wastes (bio-waste) in comparison to complete BGRs, nitrification inhibitor, agricultural BGRs, mineral fertilizer and unfertilized plots as control. The experiment was set up as a randomized block design on silt loam Cambisol. Biogas residues from four biogas plants were evaluated. Plants per m², ears per plant, grains per ear and thousand grain weight (TGW) were measured at harvest. Fertilization with BGRs resulted in similar biomass yields compared with mineral fertilizer. Mineral fertilizer (71 dt/ha) and plots fertilized with liquid fraction (59–62 dt/ha) indicated a trend to higher yields than solid fraction or complete BGR due to its high ammonia content. Liquid fractions and fraction with nitrification inhibitor induced fewer plants per m² than corresponding solid and complete variants due to a potential phytotoxicity of high NH_4_-N concentration during germination. However, barley on plots fertilized with liquid fraction compensated the disadvantages at the beginning during the vegetation period and induced higher grain yields than solid fraction. This was attributable to a higher number of ears per plant and grains per ear. In conclusion, BGRs from biodegradable municipal and industrial wastes can be used for soil fertilization and replace considerable amounts of mineral fertilizer. Our study showed that direct application of the liquid fraction of BGR is the most suitable strategy to achieve highest grain yields. Nevertheless potential phytotoxicity of the high NH_4_-N concentration in the liquid fraction should be considered.

## Introduction

The government of Germany enacted legislation to increase the percentage of energy from renewable sources like solar, wind, and bioenergy to 60% of the total energy consumption by 2050 [1]. At present, bioenergy, with a share of 61.8%, is the most important renewable energy source [2]. Bioenergy is an essential component due to its broad range of applications and its storage capacity. One of the bioenergy sector’s key technologies is the conversion of organic sources to biogas via anaerobic digestion [3]. The advantage of biogas is that it can be produced from nearly all kinds of biological feedstocks, e.g. plant biomass, animal manure, industrial organic waste and organic household waste [4–6]. The production of energy plants, especially maize, however has caused competition between food, fodder and energy production on arable land. One possible way to address the competition problem is by using other biogenic substrates like organic wastes.

Most of the approximately 8,000 installed biogas plants in Germany are fed with energy plants and animal excrements, while 8% use organic wastes like source-separated household waste, food waste, kitchen waste, sewage sludge and green waste (further bio-waste) [7].

The average production of biogas from 1 t FM bio-waste is 120 Nm³ [2].This value lies between that for animal excrements and crop silages (maize, grass, rye), indicating that such wastes are suitable substrates for biogas production [2]. In terms of the usage of organic residues and wastes it must be taken into consideration that in Germany nearly all waste materials are currently used in well-established utilization processes and only few residues and wastes are not completely utilized [8]. Residues from agriculture and agroindustry are typically used for animal feeding, compost and biogas production, whereas wastes from municipal collection and wastewater treatment are mostly used in incineration and composting plants [8]. As a consequence, the utilization of wastes for energy production is in the majority of cases in strong competition with existing utilization routes. However, there is potential for increasing the efficiency of the usage of wastes. For example, bio-wastes could first be used for biogas production with the non-fermented residues then being composted [9]. Further BGRs can be used as soil amendments due to their high plant available nutrient (N, P, K) contents and considerable amount of residual organic carbon [10–12]. An additional benefit of such a usage chain is the reduction of the amount of organic waste landfilled. [10, 13, 14]. The sustainability of the usage chain requires that the BGRs are reused without any negative environmental impacts [15].

Studies on fertilizer value and environmental impacts of BGRs were primarily conducted in the laboratory. Field studies investigating the effect of the application of BGRs on crop yields are scarce. This applies especially to separated BGRs from bio-waste.

Tambone et al. [12] and Odlare et al. [10] evaluated the fertilizing effect of a large number of unseparated organic waste products inclusive household wastes. The authors found, that unseparated BGRs had a good fertilizing properties due to the high content of plant available nutrients (NPK). Furthermore, unseparated BGRs can improve soil structure and water holding capacity and provide other advantages such as greater microbial stability and hygiene compared with untreated waste [10, 16, 17]. With the very few exceptions of cases involving feedstock with very high C/N ratio, the literature on the short-term effects of BGR on soil properties has consistently noted the improvement of the quality of soils amended with anaerobic BGRs [18].

Haraldsen et al. [19] studied the fertilizing effect of separated BGRs from source-separated household wastes especially on barley and recommended liquid BGR as a fertilizer for cereal production. De la Fuente et al. [20] evaluated separated co-digested cattle slurry and suggested a solid-liquid separation followed by composting of the solid fraction and soil application of the liquid fraction as the most suitable strategy for agricultural purposes.

Nevertheless, so far no study has compared the effects of utilizing different types of separated and unseparated BGRs from bio-wastes on yields and yield formation. There is also no data available on the fertilizing effect of separated BGRs from bio-wastes in combination with a nitrification inhibitor.

We therefore compared the fertilizing performance of separated and unseparated BGRs from different sources in a field experiment with spring barley. The hypothesises are: 1) separated BGRs sourced from biodegradable household and industrial wastes result in similar yields to those obtained by mineral fertilizers; 2) the liquid fraction of BGRs results in higher yields than the solid fraction due to its high nitrogen (N) availability and 3) usage of nitrification inhibitor leads to higher yields compared to the same biogas residue without inhibitor.

## Material and Methods

### Biogas Residues

The biogas residues (Table 1) were collected from four large-scale biogas plants. Biogas plant 1 utilizes the organic fraction of source separated household waste, green waste and catering waste. The substrate is wet digested for 20 d at 55°C. Biogas plant 2 uses dry digestion as a processing method for 25 d at >45°C. The source of the feedstock is the organic fraction of separated household waste and catering waste. In biogas plant 3 sewage sludge, catering waste, expired food and animal by-products were sanitized at 70°C and wet digested at 37–40°C for 25 d. All organic waste processing biogas plants had a thermophilic treatment over 55°C to provide epidemic hygienically harmless products. The fourth biogas plant is an agricultural plant where 90% cattle manure and 10% maize and grass silage are used. The biogas residues from waste material processing biogas plants are separated into a solid and a liquid fraction. Liquid fractions from biogas plants 1 and 2 are usually treated and disposed of in a sewage system. Solid fractions are usually composted. The liquid fraction from biogas plant 3 was used in combination with wheat straw and a nitrification inhibitor Piadin®. Biogas residue from the agricultural biogas plant was utilized without separation. Mineral fertilizer calcium ammonium nitrate was used to compare the fertilizing performance of BGR.

**Table 1:** Chemical properties of used BGRs and mineral fertilizer. TKN = total Kjeldahl nitrogen; NH_4_-N = ammonia nitrogen, FM = fresh matter, DM = dry matter, oDM = organic dry matter, BGP = biogas plant.

BGP	Biogas residue	DM (%FM)	oDM (%DM)	TKN (%DM)	NH_4_-N (%DM)	NH_4_-N from TKN (%)
1	complete residue (C1)	1.1	52.8	12.8	1.6	12.4
	liquid fraction (L1)	0.9	52.7	15	8.8	58.5
	solid fraction (S1)	35.8	69.6	1.6	0.04	2.5
2	complete residue (C2)	19.5	57.6	3.5	1.7	49.9
	liquid fraction (L2)	14.4	51.8	4.8	1.5	30.5
	solid fraction (S2)	38.8	66.8	1.8	0.03	1.7
	composted solid fraction (CS2)	52.9	54	1.4	0.02	1.8
3	liquid fraction (L3)	6.5	56.9	12	1.7	14.3
	liquid fraction + Piadin (L3+P)	6.5	56.9	12	1.7	14.3
	liquid fraction + wheat straw (L3+WS)	6.5	56.9	12	1.7	14.3
4	dairy farm biogas residue (F)	6.5	56.7	12	0.7	6.1
	mineral fertilizer (M)			27	13.5	50

### Site Characteristics and Experimental Design

The experiment was carried out at the ca. 5,000 m² experimental field of BioChem agrar GmbH in Motterwitz, Saxonia, Germany (51°11’46.63”N, 12°52’55.15”E). The long-term mean annual temperature is 8.8°C and the long-term mean annual precipitation is 641 mm.

**Fig 1:**
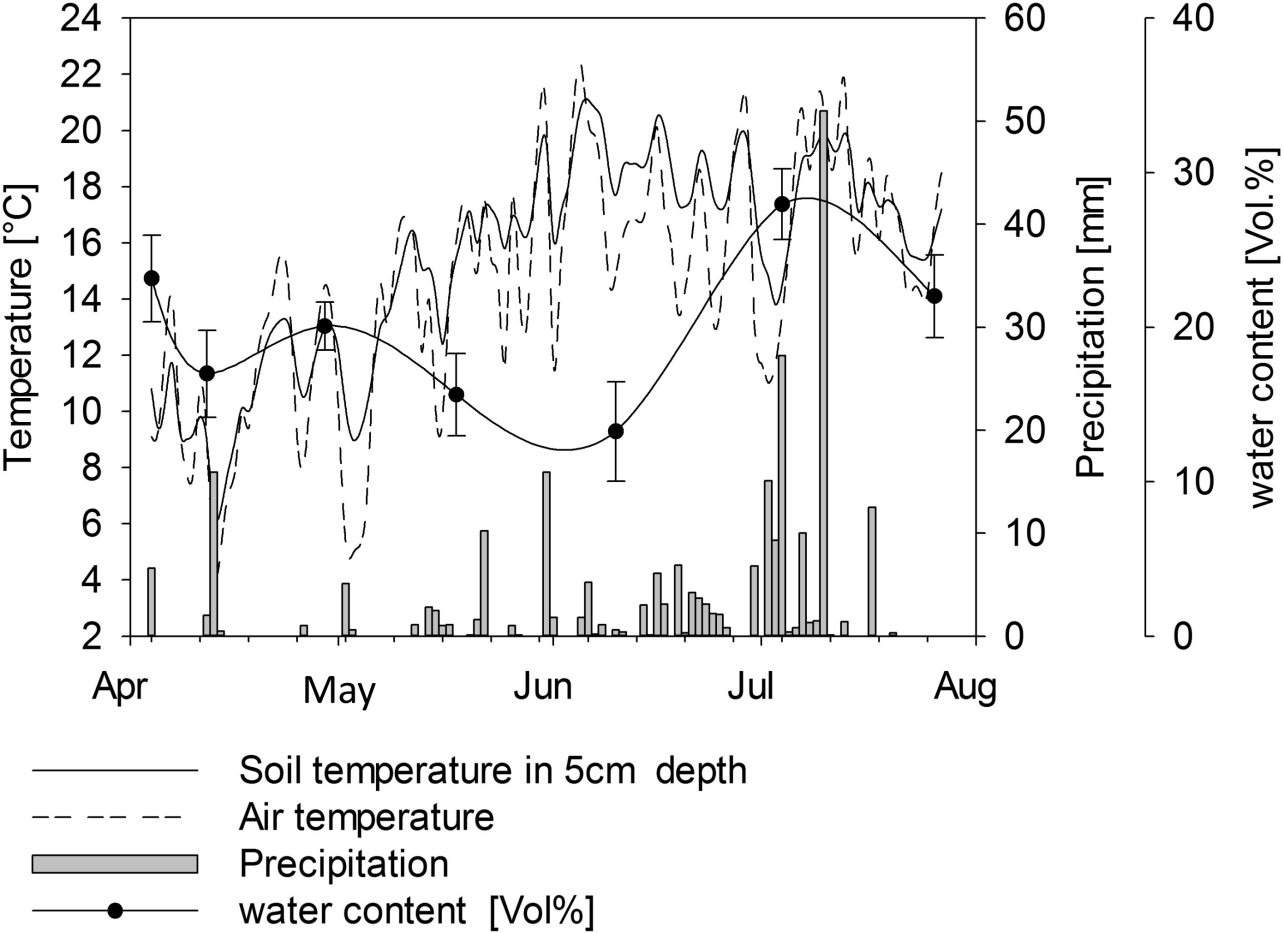
Weather conditions and soil water content during the experiment.

The growing period was from 04 April to 27 July 2011. The mean temperature during the experiment was 14.5°C (Fig 1). Total precipitation was 253.5 mm. The soil at the experimental site derived from 2 m thick loess layer and is classified as Gleyic Cambisol according to the world reference base classification system [21]. The soil (6.6% sand, 76.4% silt, and 17% clay) had a plant available water capacity of between 21.4% and 26.2%.

**Fig 2:**
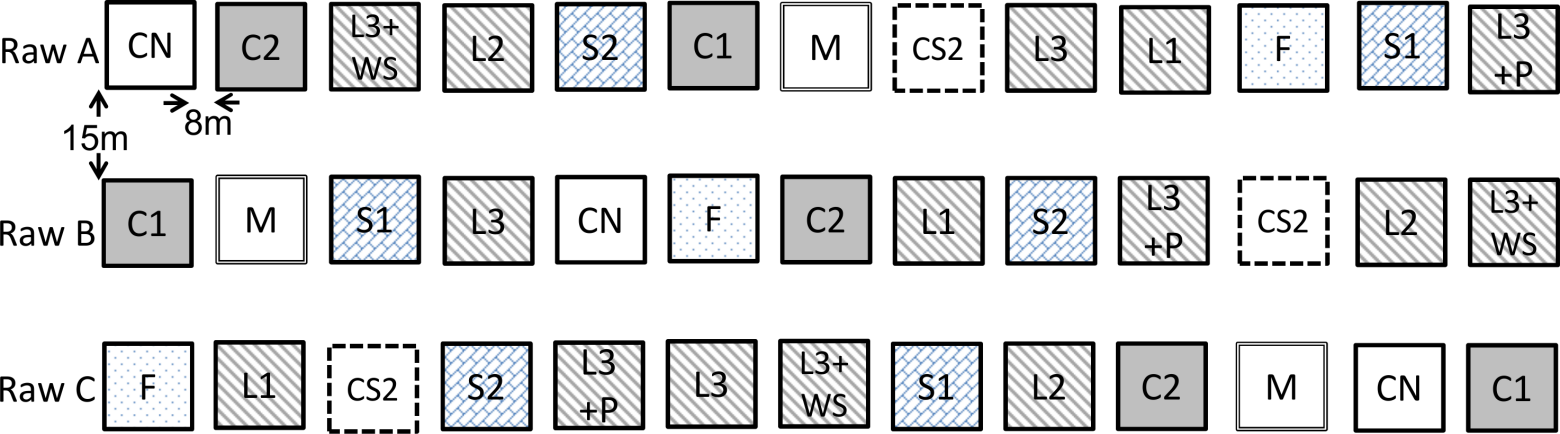
Experimental design. Abbreviations are explained in Table 1.

Biogas residues (Table 1), mineral fertilizer and unfertilized control plots (CN) were distributed in a randomized block design with three replications and an amended plot size of 4 m² (2*2 m) (Fig 2). Distances between the amended plots were 8 m and 15 m (Fig 2).

All organic and inorganic fertilizers were applied on 4 April 2011 at a rate corresponding to 65 kg N per ha. The amount of fertilizer applied was based on total Kjeldahl nitrogen (TKN) for BGR and on mineral N for mineral fertilizer. Biogas residues were spread over the soil surface manually. After application the organic materials were immediately incorporated into the soil (10 cm depth) with a rotary cultivator. Mineral fertilizer was not incorporated into the soil. Spring barley (*Hordeum vulgare L*.*)* cultivar “Laverda” was sown on 13 April using 450 seeds per m^2^ over the entire 5,000 m² field.

### Soil Sampling

Soil samples (0–20 cm) were collected before BGR application on 04.04.2011 and after application on the 12.04.2011, 29.04.2011, 18.05.2011, 10.06.2011, 04.07.2011 and 26.07.2011. One composite soil sample per plot consisting of 15 individual samples was packed in a portable cooling box in the field. For ammonium and nitrate analyses, the field moist samples were frozen moist (-20°C) upon arrival at the laboratory. For particle distribution, pH, total carbon (TC) and nitrogen (TN) aliquots were air dried and sieved at 2 mm.

### Yield Formation

Plants were hand harvested on 27 July 2011 on a subplot of 0.25 m^2^ between yellow ripeness and full ripeness. Each plant was tied separately, stored in material bags and dried in the cold air compartment at 25°C for 1 week. Plants per m² as well as ears per m² were calculated from the 0.25 m² subplots. The number of grains per ear was obtained from the mean grain number of 20 randomly selected ears from each sample. The thousand grain weight (TGW) was calculated by counting and weighing 100 grains three times. Grain yield was calculated as product of the number of grains per ear, ears per m² and TGW.

### Biogas Residue, Soil and Plant Analysis

Samples of BGR were analyzed within 24 h of collection. The BGR pH was measured potentiometrically directly in the residue. Solid fractions were diluted with distilled water (1:10) prior to measurement. The dry matter of BGR was measured gravimetrically after drying at 105°C [22] and organic dry matter was estimated after heating at 550°C [23]. Ammonium N was measured photometrically [24]. Total Kjeldahl nitrogen was measured after titration [25].

The soil pH (1:2.5; CaCl_2_) was measured potentiometrically with a glass electrode (METTLER TOLEDO, SevenEasy). Particle size distribution was analyzed according to DIN ISO 11277 [26]. The soil water content was determined gravimetrically after the samples were dried at 105°C for 24 h.

Dried and ground soil, straw and grain samples were used for measurement of TC and TN concentrations using the elementary analyzer (elementar, Vario EL).

The proof of inorganic carbon with 10% HCL showed an absence of lime in the soil. Thus TC is equivalent to the total soil organic carbon. Total N uptake was calculated as a sum of straw N and grain N. Inorganic N (N_min_) was calculated as the sum of NH_4_-N and NO_3_-N. Ammonium N and N0_3_-N concentrations in soil samples were measured photometrically (MERCK SQ 118).

### Data Treatment

Means and standard deviations were calculated. The effects of different treatments on spring barley yield formation and soil chemical parameters were analyzed by a one-way ANOVA. Normal distribution was tested with a Shapiro test. Least significant difference t-Test (LSD) was used to compare mean values and to assess the significance of the differences between mean values. Effects were considered significant for *p* < 0.05. All statistical analyses were performed using R version 3.0.1 (The R Foundation for Statistical Computing, 2013).

## Results

### Composition of Biogas Residues

The pH in all BGRs was mostly similar (Table 1). Dry matter differed due to the wet or dry digestion process as well as due to the liquid or solid separation. After separation the N concentration was higher in the liquid than in the solid fraction.

### Effect on Soil Properties

**Fig 3:**
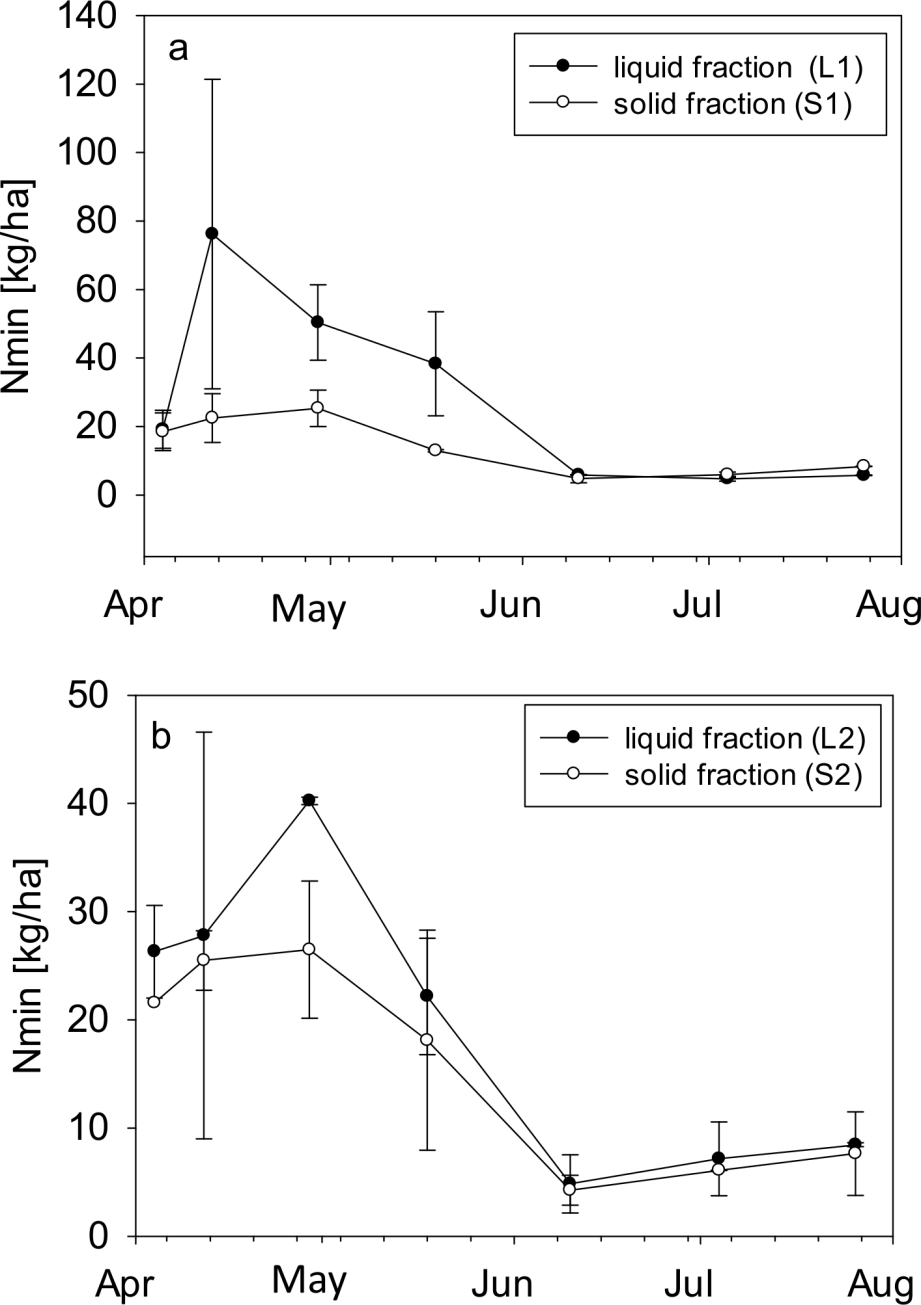
Temporal sequence of mineral nitrogen (Nmin) in soil (0-20cm depth) during the vegetation period in plots fertilized with a) L1 = liquid fraction of BGR from biogas plant 1 and S1 = solid fraction of BGR from biogas plant 1 and b) with L2 = liquid fraction of BGR from biogas plant 2 and S2 = solid fraction of BGR from biogas plant 2.

Chemical parameters of the soil (Table 2) were homogeneous throughout all experimental plots at the beginning of the experiment. Total C and TC/TN ratio did not change during the experimental period. The pH tended to decrease, but was not statistically significant. After harvesting, the concentration of N_min_ in soil decreased by about 50% due to nitrogen plant uptake. Soil N_min_ in plots fertilized with liquid fraction increased after BGR application and decreased over the vegetation period (Fig 3 and S1 Table). Soil N_min_ in plots fertilized with the solid fraction of BGR remained low throughout the observation period (Fig 3). We used only BGRs from plant 1 and plant 2 to illustrate the differences between the solid and the liquid phase of BGRs; the same comparisons could not be drawn for plants 3 and 4 because both liquid and solid fractions of BGRs from these plants would be required for this and these were not available. For this reason we did not present data from plants 3 and 4. Soil water content fluctuated according to precipitation (Fig 1), without any difference between the experimental plots. The water supply among the plots was equal and without significant differences.

**Table 2:** Soil properties before fertilization and after harvesting. mean ± sd = mean value ± standard deviation, n.a. = sd is not possible, TC = total carbon, TN = total nitrogen, N_min_ = mineral nitrogen.

	before fertilization				after harvest			
	pH	TC (%)	C/N	Nmin (kg/ha)	pH	TC (%)	C/N	Nmin (kg/ha)
	mean ± sd	mean ± sd	mean ± sd	mean ± sd	mean ± sd	mean ± sd	mean ± sd	mean ± sd
C1	5.9 ± 0.2	1.3 ± 0.1	9.2 ± 0.1	29.5 ± 10.8	5.6 ± 0.2	1.3 ± 0	9.1 ± 0.4	6.7 ± 0
L1	5.9 ± 0.3	1.1 ± 0.1	9.2 ± 0	19.2 ± 5.5	5.6 ± 0.4	1.1 ± 0.1	9.4 ± 0.1	5.8 ± 0.1
S1	5.8 ± 0.3	1.1 ± 0.1	9 ± 0.5	18.5 ± 5.5	5.6 ± 0.3	1.2 ± 0.1	9.3 ± 0.2	8.3 ± 0
C2	5.8 ± 0.3	1.2 ± 0.1	9.3 ± 0.1	28.4 ± 0	5.5 ± 0.3	1.2 ± 0.1	9.4 ± 0.3	8.3 ± 1.3
L2	5.5 ± 0	1.3 ± 0.1	9.2 ± 0.1	26.3 ± 4.3	5.8 ± 0	1.3 ± 0.2	9.2 ± 0	8.5 ± 0.2
S2	5.7 ± 0.2	1.1 ± 0.2	9.3 ± 0.2	21.6 ± n.a.	5.5 ± 0.2	1.1 ± 0.2	9.1 ± 0.3	7.6 ± 3.9
CS2	5.9 ± 0.4	1.2 ± 0.3	9.2 ± 0.3	25.5 ± 6.6	5.9 ± 0	1.2 ± 0.3	9.2 ± 0.4	9.3 ± 1.6
L3	6.2 ± 0	1.1 ± 0.2	9.3 ± 0.3	22.4 ± 2.6	5.6 ± 0.3	1.1 ± 0.2	9.2 ± 0.5	7.8 ± 3.8
L3+P	6.1 ± 0	1.1 ± 0.2	9.4 ± 0.3	28 ± 1.1	5.6 ± 0.3	1.1 ± 0.2	8.6 ± 0.5	7.8 ± 0
L3+WS	5.8 ± 0.4	1.2 ± 0.2	8.8 ± 0.4	26.6 ± 2.1	5.6 ± 0.3	1.2 ± 0.2	9.3 ± 0	9.8 ± 0.6
F	5.9 ± 0.4	1.3 ± 0.3	9.3 ± 0.3	24.6 ± 0.6	5.6 ± 0.4	1.1 ± 0.1	9.3 ± 0.3	6.9 ± 2.1
M	5.9 ± 0.2	1.3 ± 0	9.2 ± 0.1	30.6 ± 6.1	5.6 ± 0.1	1.3 ± 0	9.3 ± 0.2	8.8 ± 0.1
CN	6.2 ± 0.1	1.2 ± 0.2	9.4 ± 0.1	29.4 ± 5.5	5.9 ± 0.2	1.2 ± 0.2	9 ± 0.3	7.9 ± 0.1

### Effects on Yields and Yield Formation

All applied fertilizers induced significantly higher yields of barley grain and straw than the unfertilized control (Table 3). Nonetheless, plots which were fertilized with mineral fertilizer showed a trend towards 15% higher yields than BGR fertilized plots. Fertilizer treatments with high grain yields did not necessarily lead to a high straw yield (Table 3). Plots which were amended with liquid fraction of BGR (L1, L2, L3) tend to produce a higher grain yield than plots amended with solid fractions (S1, S2), composted (SC2) or complete BGR (C1, C2, F). Composted and solid fractions (CS2, S2) gave the same yield. Grain N content was higher in plots fertilized with S2. Addition of the nitrification inhibitor did not influence biomass and grain yield.

**Table 3:** Yield formation, grain and straw yields, N content in grain and aboveground biomass N uptake. Biogas residue abbreviations are explained in Table 1. mean ± sd = mean value ± standard deviation, LSD = Least Significant Difference t-Test. Means with the same letter are not significantly different.

BGR	plants/m²		ears/m²		ears/plant		grains/ear		TGW (g)		yield (dt/ha)		straw (g/m²)		N grain (%)		N uptake (kg/ha)	
	mean ± sd	LSD	mean ± sd	LSD	mean ± sd	LSD	mean ± sd	LSD	mean ± sd	LSD	mean ± sd	LSD	mean ± sd	LSD	mean ± sd	LSD	mean ± sd	LSD
C1	394 ± 70.7	ab	914 ± 144.2	a	2.3 ± 0.1	b	14.9 ± 0.3	ab	42.1 ± 1	abc	57.1 ± 6.5	ab	861.1 ± 82.1	ab	1.8 ± 0	ab	147.9 ± 22.5	ab
L1	298 ± 2.8	ab	812 ± 67.9	ab	2.7 ± 0.3	ab	16.3 ± 0.2	ab	46.1 ± 5.7	abc	58.5 ± 3.8	a	655.1 ± 59.4	ab	1.8 ± 0.3	ab	152.3 ± 40.2	ab
S1	350.7 ± 97.8	ab	897.3 ± 130.1	a	2.6 ± 0.5	ab	14.9 ± 1.6	ab	41.6 ± 2.9	bc	55.1 ± 4	ab	800.2 ± 41.7	ab	1.8 ± 0.2	ab	136.7 ± 46.3	ab
C2	384 ± 41.8	ab	969.3 ± 40.5	a	2.5 ± 0.2	ab	15.8 ± 0.6	ab	39.3 ± 1.4	bc	60.3 ± 1.9	a	877.5 ± 102.4	ab	1.6 ± 0.2	ab	120.6 ± 56.2	ab
L2	354 ± 42.4	ab	868 ± 67.9	ab	2.5 ± 0.1	b	15.7 ± 1.9	ab	45.2 ± 2.6	ab	62.3 ± 1.3	a	795.1 ± 89.2	ab	1.7 ± 0.2	b	128 ± 66.4	ab
S2	350.7 ± 70.5	ab	952 ± 188.6	a	2.8 ± 0.5	ab	14.6 ± 2.1	ab	41 ± 6.6	bc	55.9 ± 6.5	ab	855.7 ± 200.2	ab	1.9 ± 0.1	a	158.6 ± 11.9	ab
SC2	362.7 ± 22	ab	856 ± 8	ab	2.4 ± 0.2	b	15.4 ± 0.7	ab	42.2 ± 3.3	abc	55.5 ± 5	ab	782.3 ± 67.2	b	1.6 ± 0.2	b	122.7 ± 19.7	ab
L3	374.7 ± 10.1	ab	885.3 ± 181.5	a	2.4 ± 0.5	b	14.7 ± 0.8	b	46.6 ± 1.6	ab	60.1 ± 10.4	a	871.5 ± 245.1	b	1.6 ± 0.3	ab	140.9 ± 23.1	ab
L3+P	258.7 ± 96.1	b	876 ± 43.3	ab	3.8 ± 1.6	a	17.1 ± 0.2	a	41.4 ± 3.3	abc	61.7 ± 2	a	711.9 ± 170.7	ab	1.6 ± 0.1	ab	134.1 ± 6.8	ab
L3+WS	336 ± 170.3	ab	785.3 ± 119.5	ab	2.9 ± 1.6	ab	15.6 ± 2	ab	40.2 ± 6.6	abc	48.8 ± 9.9	ab	646.6 ± 260.9	ab	1.7 ± 0.2	ab	100.5 ± 11.7	ab
F	420 ± 32	a	885.3 ± 224	ab	2.1 ± 0.7	b	15.2 ± 0.4	ab	41.1 ± 2.9	abc	56 ± 19	ab	723.9 ± 208.2	a	1.7 ± 0.4	ab	160.7 ± 80	ab
M	480 ± 118.8	a	950 ± 291.3	a	2 ± 0.1	b	16 ± 1.2	ab	46.6 ± 2	ab	71 ± 20.7	a	957.4 ± 213.2	ab	1.8 ± 0.1	ab	173.2 ± 77.6	ab
CN	336 ± 45.1	ab	644 ± 74.9	bc	1.9 ± 0.2	b	14.2 ± 1.7	b	40.8 ± 3.2	bc	37 ± 2.6	b	483 ± 38.3	b	1.5 ± 0.1	b	77.6 ± 4.2	b

Plots with highest yields did not necessarily have the best performance in each category of the yield formation. Liquid fractions led to fewer plants per m² at the beginning of the vegetation period in comparison to solid and complete BGR. Later these variants compensated this disadvantage with more ears per plant and grains per ear compared to the other treatments. Differences in TGW among all treatments are not significant. Nevertheless, the TGW of the liquid variants tended to be higher compared with other treatments. Within biogas plant 3, L3 and L3+WS led to 50% more plants per m² than L3+P. During the vegetation period L3+P plants compensated the low number of plants with a high number of ears and a high number of grains per ear. In general, plots fertilized with M had the best performance in every yield formation category.

## Discussion

### Biogas Residue Properties

The BGRs tested in this study were produced in different biogas plants operating with different digestion techniques and substrates. The composition of the different BGRs can vary greatly depending on the feedstock used for co-digestion and the process characteristics [5, 27]. The result of different input or different treatment and separation technique can also be different N concentrations among the BGRs [28, 29]. The fermentation process increases the availability of N (NH_4_-N) due to the breakdown of organically bound N during the anaerobic process [9, 16, 28]. Measured NH_4_-N proportion of total N in liquid fraction was between ca. 14% and 59% and in solid fraction between 2% and 3%. This is because after the separation process NH_4_-N almost entirely migrates into the liquid fraction [18, 28, 30]. Like compost, the solid fraction is a poor source of N due to the low content of mineral N and low mineralization, which is in agreement with our results [13, 31]. Thus the liquid fraction of BGRs from bio-wastes can replace substantial amounts of mineral N and can substitute mineral fertilizer. However, increased NH_4_-N concentration in digested slurries compared to undigested slurries does not necessarily guarantee improved uptake efficiency of slurry N or increased savings in fertilizer N [32]. Nitrification of the ammonium N in the BGR can cause significantly increased nitrate leaching [19]. Furthermore, alkaline pH and high NH_4_-N concentration of BGRs may result in N losses due to NH_3_ volatilization [33–35]. However, a higher ammonia volatilization potential compared with undigested slurry does not necessarily result in higher emissions since the lower solid content and reduced viscosity lead to a better infiltration characteristics [6]. Results in the literature on the effect of anaerobic digestion on ammonia volatilization after field application therefore are inconsistent [36].

### Effects on Soil Properties

Biogas residues have an alkaline pH. Al-Juhaimi et al. [37] and de la Fuente et al. [20] reported that the pH of the soil decreased after application of the liquid fraction from BGRs and increased after solid fraction application. In our study, biogas residues did not affect soil pH despite values above pH 8 in BGRs. This could be attributed to the high buffer capacity of the clay minerals in the silty-loam soil.

Contradicting information is given in literature about C and N change in soil after BGR application. Some authors showed that land application of BGRs had short-term benefits in terms of improving SOM stock due to the addition of the organic matter with this material [20, 38, 39]. In contrast, but similar to our study, Bachmann et al. [40] reported that, even after 3 years, there was no change of organic C content in soil. The effect on soil N and C turnover and in contributions to soil C storage of digested materials is small compared to the amount of N and C already residing in the soil and therefore difficult to quantify over shorter time spans [11]. The mean TC of our soil was 1.2%. With BGRs only 0.02–0.18% organic matter was additionally applied in the upper 10 cm.

### Effects on Yields and Yield Formation

Contrasting results for the effect of BGRs on yield are reported [32]. Those research results can be grouped into three categories of performance: (a) performances similar to unfertilized controls [13], (b) performances similar or higher than undigested feedstock [16, 35] and (c) performances equal or better than mineral fertilizers [41]. None of the BGRs used in the present study showed a negative effect on barley yield. This is in line with results reported by others [41, 42]. The yields measured in our experiment correspond to average yields (59 dt/ha) for the “Landkreis Leipzig” study region in 2011 [43]. Fertilization with BGRs was based on TKN which is not completely directly available to plants. Nevertheless, no significant differences in the grain yields of BGR- and M-fertilized plots were found, hence BGRs have a high fertilizing potential.

One important plant nutrient is mineral N, which is generally the limiting factor for crop growth [44]. In the literature, high fertilizing potential is associated with NH_4_-N content which is immediately plant available after application [45]. The mineral fertilizer N is directly plant available too. A large proportion of N in liquid variants was directly plant available as well. In accordance with that, soil N_min_ in L2 variants was high at the beginning of the vegetation period and decreased until the end of the vegetation period due to plant uptake. Nitrogen in solid variants has to be mineralized before plants can take it up. Soil N_min_ in plots fertilized with S2 was low throughout the observation period. The plant availability of N in these plots started only during the grain filling phase. As a result, grain N of solid variants was equal or significantly higher compared to liquid variants.

In some cases factors like soil water content and temperature may have a greater effect on the uptake of nutrients than the amount of nutrients applied as fertilizer [46]. Under dry weather conditions barley yield did not increase, even if soil N resources were high [47]. Since water supply did not differ significantly among the plots, fertilization is the single influencing factor on yield formation in this case.

The grain yield of cereals is a product of three basic compounds: ears per m², grains per ear and TGW [46]. Ears per m² is a product of plants per m² and ears per plant. The number of plants per m² depends on sowing and initial growth conditions. The number of ears per plant depends on growth conditions during tillering. Spring barley was sown at a constant number of 450 seeds per m² over the whole field and produced a number of plants per m² which is in line with the literature [48]. Nevertheless, the liquid fractions L1 and L2 led to fewer plants per m² than corresponding solid and complete variants. The reason for this could be a higher NH_4_-N concentration, which can reflect a potential phytotoxicity of organic products during germination [45, 49, 50]. L3+P gave rise to fewer plants per m² than L3 and L3+WS. Nitrification was inhibited, NH_4_-N concentration remained high, and induced higher damage through ammonia toxicity compared to variants without Piadin^®^. Nevertheless plots fertilized with liquid fraction compensated the initial disadvantages over the vegetation period and induced higher grain yields.

One result of our study was that fertilization with liquid variants in comparison to other variants tended to result in a higher number of grains per ear, which is the most common limiting yield component for wheat and barley, and is dependent on N supply during tillering [51]. Average value for grains per ear in Germany is 17.9 grains [52]. Other authors have stated a higher number of grains per ear of between 18.1 and 28 grains [47, 48, 50]. In our study the higher grain yield was attributable to a higher number of ears per plant and grains per ear, which is in line with Schittenhelm and Menge-Hartmann [50].

The thousand grain weight on plots fertilized with liquid fractions was higher than on corresponding variants as well. Grains achieved a TGW which is average for spring barley in Germany (45 g) [52]. Since TGW depends on temperature, moisture and mineral nutrient availability at grain filling, the N supply of liquid fractions was adequate [46]. In addition liquid variants achieved a high TGW without a late application of N which affects the weight of grains [46].

### Risks Associated with Land Application

The relatively high mineral N content, up to 80% of which was present in the form of NH_4_-N, indicates a high potential for N losses during BGR handling and application [32]. High pH and NH_4_-N concentrations are conditions that favour NH_3_ emissions [18]. Inappropriate storage or application of BGRs represent a risk of air and water pollution due to possible gaseous nitrogen emissions and/or nutrient leaching and runoff into surface and ground waters [18]. Land application of BGRs as fertilizer is not risk-free, since it may result in soil contamination including physical contaminants such as plastics, glasses and stones as well as chemical contaminants such as phytotoxic compounds, pathogens and heavy metals [18].

## Conclusions

The initial short-term fertilization effect of soil treated with BGRs from bio-wastes is similar to agricultural BGRs and mineral fertilizer. Thus BGRs can replace a considerable amount of mineral fertilizer. The nitrification inhibitor did not impact the grain yield. Biogas residues affected the yield formation. Liquid fraction alone or in combination with nitrification inhibitor induced fewer plants per m² than corresponding solid and complete variants or variants without the inhibitor. This might be explained by the high potential phytotoxicity of NH_4_-N. Nevertheless, barley compensated the initial disadvantages over the vegetation period.

To date liquid fractions from biogas plant 1 and 2 were disposed of through the sewage system after treatment. Our study showed that direct application of the liquid fraction is the most suitable strategy to achieve highest yields. It provides agricultural benefits and helps to close nutrient cycles. However, it can cause damage due to NH_4_-N phytotoxicity. Solid fractions could be composted before being used as a soil conditioner. This would reduce transport costs due to the resulting lower volume and facilitate its addition to soil. Furthermore, composting reduces water content and odor emissions due to the reduction of volatile compounds and potential phytotoxicity, and also contributes to the elimination of pathogens. Nevertheless during a land application potential physical, chemical and biological soil contaminations as well as atmospheric and water pollutions should be considered.

## Supporting Information

S1 Table Temporal sequence of mineral nitrogen (Nmin) in soil (0-20cm depth) during the vegetation period in plots fertilized with a) L1 = liquid fraction of BGR from biogas plant 1 and S1 = solid fraction of BGR from biogas plant 1 and b) with L2 = liquid fraction of BGR from biogas plant 2 and S2 = solid fraction of BGR from biogas plant 2.
(XLSX)
